# Insight into Gentisic Acid Antidiabetic Potential Using In Vitro and In Silico Approaches

**DOI:** 10.3390/molecules26071932

**Published:** 2021-03-30

**Authors:** Hamza Mechchate, Imane Es-safi, Omkulthom Mohamed Al kamaly, Dalila Bousta

**Affiliations:** 1 Laboratory of Biotechnology, Environment, Agrifood, and Health, Department of Biology, University of Sidi Mohamed Ben Abdellah, FSDM-Fez 30050, Morocco; Imane.essafi1@usmba.ac.ma (I.E.-s.); boustadalila@gmail.com (D.B.); 2 Department of Pharmaceutical Sciences, College of Pharmacy, Princess Nourah bint Abdulrahman University, Riyadh 11564, Saudi Arabia; omalkmali@pnu.edu.sa

**Keywords:** gentisic acid, in silico, molecular docking, in vitro, DPP4, PTP1B, FFAR1, AldR, GP, α-amylase, PPAR-γ, α-glucosidase

## Abstract

Numerous scientific studies have confirmed the beneficial therapeutic effects of phenolic acids. Among them gentisic acid (GA), a phenolic acid extensively found in many fruit and vegetables has been associated with an enormous confirmed health benefit. The present study aims to evaluate the antidiabetic potential of gentisic acid and highlight its mechanisms of action following in silico and in vitro approaches. The in silico study was intended to predict the interaction of GA with eight different receptors highly involved in the management and complications of diabetes (dipeptidyl-peptidase 4 (DPP4), protein tyrosine phosphatase 1B (PTP1B), free fatty acid receptor 1 (FFAR1), aldose reductase (AldR), glycogen phosphorylase (GP), α-amylase, peroxisome proliferator-activated receptor gamma (PPAR-γ) and α-glucosidase), while the in vitro study studied the potential inhibitory effect of GA against α-amylase and α-glucosidase. The results indicate that GA interacted moderately with most of the receptors and had a moderate inhibitory activity during the in vitro tests. The study therefore encourages further in vivo studies to confirm the given results.

## 1. Introduction

Recently, medicinal plants and natural products have gained more popularity in the field of drug discovery, with many active compounds studied and pharmacologically approved within the past decade [1]. The therapeutic usage of plants in the treatment of various challenging illnesses incorporates a long history of clinical use, indicating in most cases a high patient tolerance and acceptance [2]. A large number of plant species and plant-derived bioactive molecules have been screened for their medicinal use, and many of them are commercialized and constitute essential parts of the therapies used nowadays. A few relevant examples are: morphine isolated in the early 1800s by shallowly scoring the unripe seedpods of the *Papaver somniferum* poppy, aspirin isolated from *Salix alba* in 1899, paclitaxel from *Taxus brevifolia* in 1962, artemisinin in 1972, silymarin, cocaine, codeine, digitoxin, quinine and pilocarpine and many others [3,4].

Plant-derived bioactive molecules were subjected to profound studies to discover the relationship between the chemical composition and pharmacological activities, leading to (i) substantial progress in the design and (ii) synthesis of new compounds and amelioration of their pharmacokinetics and pharmacodynamics proprieties [5].

Plant biomolecules have been classified and divided following their chemical characteristics into many families and subfamilies. Among them, phenolic acids (or phenol carboxylic acids), derived from either cinnamic or benzoic or acid skeletons specify a wide association of compounds forming an aromatic ring containing hydroxyl and carboxyl substitutes. Over the past few years, a growing body of clinical research has focused on the possible impact of phenolic acids on tumor progression, cardiovascular disease and other health problems (such as inflammation, diabetes, neurological disorders) [6]. Widely distributed in vegetables and fruits and considered as secondary plant products such protocatechuic, caffeic, vanillic, sinapic, syringic, *p*-coumaric, salicylic, ferulic, p-hydroxybenzoic, gallic and gentisic acids synthesized basically from tyrosine or phenylalanine (shikimate pathway) [7]).

Gentisic acid (GA), also known as gentisate or 2,5-dihydroxybenzoic acid (2,5-DHBA) isolated initially from the root of the genus *Gentiana*, is a hydroxybenzoic acid derivative and one of the metabolites of aspirin [8]. GA is commonly found in many plants like gentian, batoko plum, kiwi, grapes, citrus, apple, bitter melon, Jerusalem artichoke, olives, sesame, avocados, and blackberries [9].

Gentisic acid has numerous pharmacological activities like antioxidant, anticarcinogenesis, hepatoprotective, antimicrobial, analgesic, neuroprotective, and cardioprotective [9]. A study conducted by Shivanagoudra et al. [10] evaluated the in vitro α-glucosidase and α-amylase activities of gentisic acid 5-*O*-β-d-xyloside and found an inhibition rate between 56% to 75%. Therefore, it is known that after ingestion, polyphenols and in order to be absorbed, the attached sugar must be removed [11], meaning that principally only the aglycone form (gentisic acid) is going to reach the circulation and exhibit its activity. This study was undertaken (i) to reveal the potential action of gentisic acid as an antidiabetic molecule using in vitro methods (ii) assessing its ability to inhibit the α-amylase and α-glucosidase activities and in silico, (iii) studying its mode of action using molecular docking against eight receptors that have a direct impact on the overall status of diabetes (dipeptidyl-peptidase 4 (DPP4), protein tyrosine phosphatase 1B (PTP1B), free fatty acid receptor 1 (FFAR1), aldose reductase (AldR), glycogen phosphorylase (GP), α-amylase (AAM), peroxisome proliferator-activated receptor gamma (PPAR-γ), and aplha-glucosidase).

## 2. Results and Discussion

### 2.1. Molecular Docking

The receptors selected for this research were strongly involved in the complications and treatments of diabetes. The criteria to choose a ligand pose are its affinity and interaction with the receptor’s active site. To gain a better insight from the results, a comparison was made with another molecule, “amentoflavone” a well-known antidiabetic molecule [12], using the same docking methodology. The affinity results are presented in Table 1 and the docking poses for amentoflavone are provided in the supplementary file.

#### 2.1.1. Protein Tyrosine Phosphatase 1B (PTP1B) 

The protein tyrosine phosphatase 1B (PTP1B) is an important regulator enzyme that interact with the insulin receptor (dephosphorylation) and at downstream signaling components [13]. Genetic evidence and multiple studies linked PTP1B to type 2 diabetes with a contribution to insulin resistance and obesity in humans [14]. In a study conducted by Elchebly et al., mice with a deficiency in PTP1B demonstrated resistance to weight gain with a marked insulin sensitivity while they were on a high-fat diet [13,15]. 

The catalytic loop is identified by the amino acids Trp179, Pro180, Asp181 [16], Gly218, Ile219, Gly220, His214, Ser216, Ala217, and Arg221 [17,18] and the active site on Cys 215 [18].

**Figure 1:**
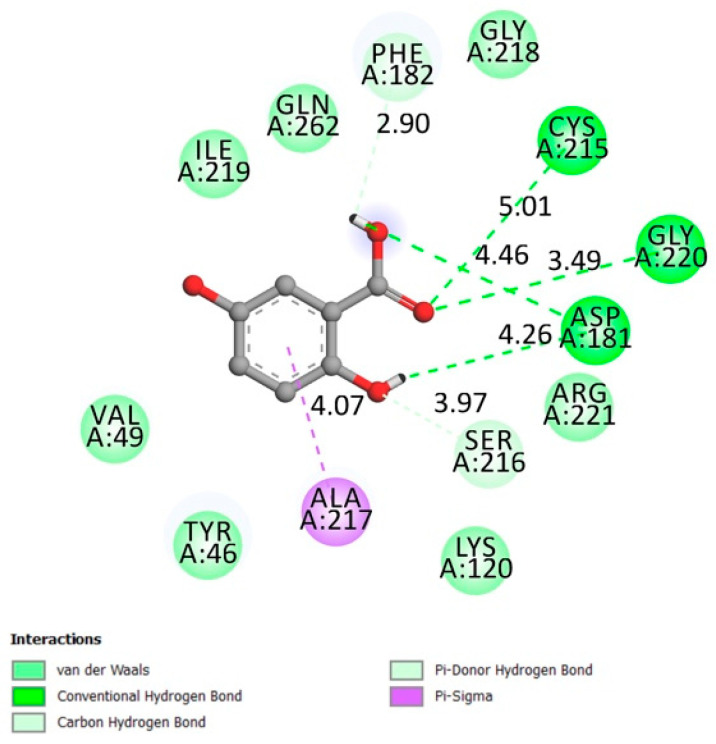
Two-dimensional (2D) scheme of the gentisic acid (GA) interactions with protein tyrosine phosphatase 1B (PTP1B) receptor.

The results described in Figure 1 indicate that gentisic acid made an interaction with the catalytic site (conventional hydrogen bonds on ASP181 and Gly220, Van der Waals bonds Arg 221, Gly218, and Ile219) and the active site (conventional hydrogen bond on Cys215) while the docking affinity was −6.1 kcal/mol. 

#### 2.1.2. Dipeptidyl-Peptidase 4 (DPP4)

Originally located on multiple cell surfaces, with various denominations such as adenosine deaminase complexing protein 2 (ADCP 2) and T-cell activation antigen CD26, dipeptidyl-peptidase 4 (DPP4) is classified as a serine exopeptidase (belongs to S9B family) whose main activity is to cleave X-proline dipeptides from the N-terminus of polypeptides such as growth factors, neuropeptides and incretin hormones (one of the most important regulators of postprandial insulin secretion) [19]. Inhibition of DPP4 provides a new possible treatment solution to type 2 diabetes mellitus as monotherapy, and it is currently available for medical applications [20]

**Figure 2:**
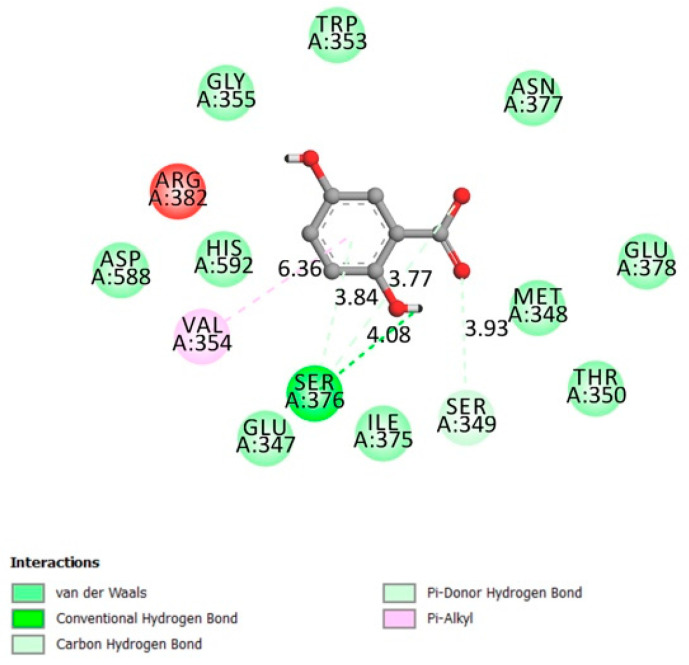
Two-dimensional scheme of the GA interactions with dipeptidyl-peptidase 4 (DPP4) receptor.

The DPP4 active site (α/β-hydrolase domain) is identified by the residues from 39 to 51 and from 501 to 706 [21,22]. Low interactions, according to Figure 2 were made between gentisic acid and DPP4 receptor in the α/β-hydrolase domain (Van der Waals bonds on His592 and Asp588), adding to this a docking affinity of −6.7 kcal/mol. This receptor’s inhibition is considered one of the type 2 diabetes promising therapy (gliptin family).

#### 2.1.3. Free Fatty Acid Receptor 1 (FFAR1)

Free fatty acid receptor 1 (FFAR1) or human G-protein coupled receptor 40 (hGPR40) is found primarily in pancreatic β-cells and some endocrine cells and can also be found in the brain [23]. With a large distribution in the pancreatic β cells, its function constitutes the recognition of the long-chain of free fatty acids and a contribution to insulin secretion [24]. The free carboxyl group of the free fatty acids binds to FFAR1 amino acids Arg183, Arg258, and Tyr2240 located near the extracellular domain [25], while the binding pocket is recognized by the amino acids Glu172, Arg183, Ser187, Tyr240, Asn241, Asn244, Arg258 and Tyr91 [26,27].

The docking results and through multiple poses did not show any kind of interaction between gentisic acid and the FFAR1 receptor.

#### 2.1.4. α-Amylase 

α-amylases are widely found in many living organisms (plants, animals, bacteria, and fungi). They are also found in human’s salivary glands, and it is secreted continuously by the pancreas into the small intestine during digestion. They are classified as amylases enzymes (alpha, beta, and gamma) that catalyze the hydrolysis of polysaccharides into monosaccharides, disaccharides, and trisaccharides, which are converted further to a glucose supply for the body’s energy [28,29]. 

Asp197, Glu233, and Asp300 constitute α-amylase active sites along with other important residues like Arg337 and 195, Trp203, 284, 58 and 59, Phe298, 265 and 295, His101, 201 and 299, Asn298, Ala307, Gly306, Tyr62, [30,31,32,33].

**Figure 3:**
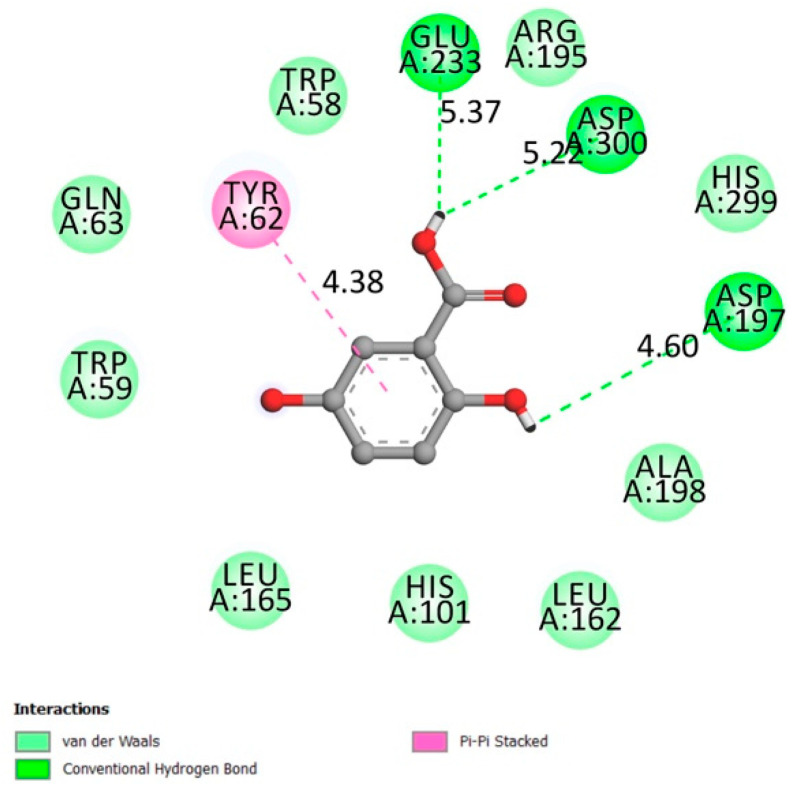
Two-dimensional scheme of the GA interactions with α-amylase receptor.

Figure 3 represents the interactions between gentisic acid and the α-amylase receptor on the catalytic residues (hydrogen conventional bond Asp300, Glu233, and Asp197) and the other confirmed important residues (Van der Waals bonds Arg 195, Trp58, and Trp59), with an affinity of −5.6 kcal/mol. 

#### 2.1.5. Peroxisome Proliferator-Activated Receptor Gamma (PPARγ) 

Peroxisome proliferator-activated receptor gamma (PPAR-γ or PPARG, glitazone receptor) is a key protein in the regulation of fatty acid storage and the metabolism of glucose, leading to increased insulin sensitivity and reduced lipotoxicity [34]. 

The PPARγ ligand-binding domain is located between the amino acids Ser289, His323, Tyr473, and His449 [35].

Analysis of the docking results and through multiple poses did not show any potential interaction between gentisic acid and the PPARγ receptor (also compared with rosiglitazone as a reference, which shows a perfect binding to the active site).

#### 2.1.6. α-Glucosidase

α-glucosidase (also known as α-d-glucosidase, maltase, glucosidosucrase, glucoinvertase, maltase-glucoamylase, α-glucopyranosidase, glucosidoinvertase, α-1,4-glucosidase, α-d-glucoside glucohydrolase, α-glucoside hydrolase) is an enzyme primarily found in the small intestine at the brush border. Its prominent role is to catalyze the hydrolysis of polysaccharides (Starch) to glucose (acts upon α(1→4) bonds) to facilitate its absorption, leading to higher levels of blood glucose [36]. 

The amino acids involved in the α-glucosidase activity are Asp404, 518, 616, 645, Trp376, 516, 613, 481 Arg600, His674, Ile441, Met519, Phe649, Leu405, and Arg672 [37,38].

**Figure 4:**
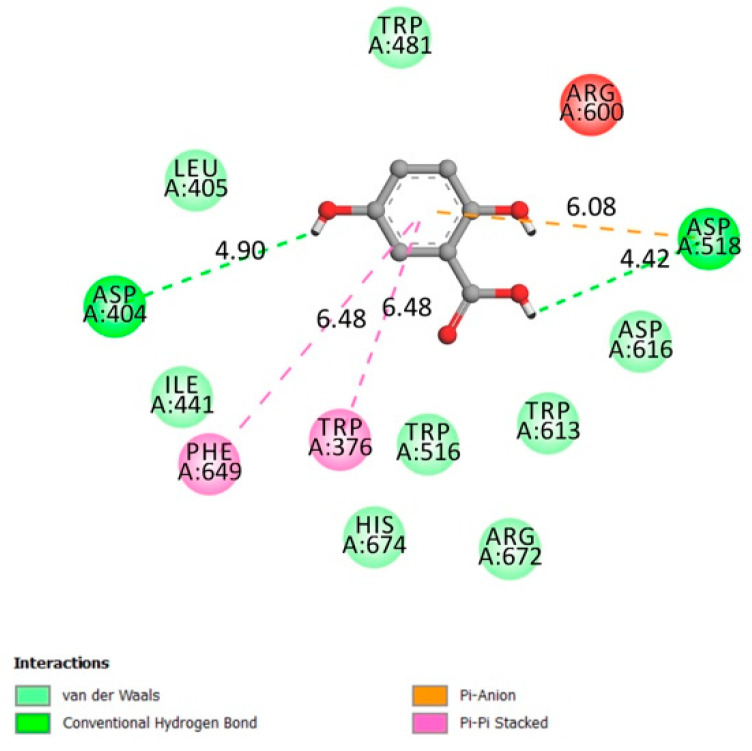
Two-dimensional scheme of the GA interactions with α-glucosidase receptor.

Figure 4 indicates that gentisic acid interacted with many amino acids related to the receptor activity (hydrogen conventional bond Asp404 and Asp518, Van der Waals bonds Trp481 Leu405 Ile441 Trp516 Arg672) with an affinity of −6.1 kcal/mol. Inhibiting such an enzyme could be beneficial in terms of reducing postprandial glycemia.

#### 2.1.7. Aldose Reductase

Located in various organs in the body like the retina, lens, Schwann cells, red blood cells, aldose reductase (AldR) is considered an essential enzyme with its role consisting of the catalyze of the NAD(P)H-dependent reduction of glucose to sorbitol through the sorbitol-aldose reductase pathway, resulting on an excessive accumulation of intracellular reactive oxygen species (ROS) in various tissues [39], causing potential multiple health complications [40].

**Figure 5:**
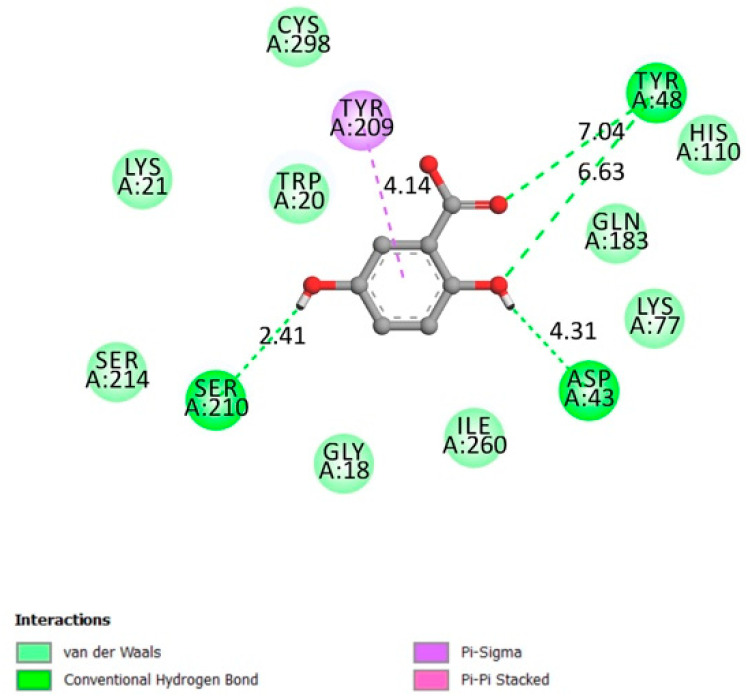
Two-dimensional scheme of the GA interactions with aldose reductase receptor.

The best pose with the best affinity (−6.9 kcal/mol) in the barrel core of the aldose reductase receptor (active site [41]) is represented in Figure 5. 

#### 2.1.8. Glycogen Phosphorylase

Glycogen phosphorylase (GP) is one of the phosphorylase enzymes that catalyze the hydrolysis of glycogen to generate glucose-1-phosphate [42]. 

**Figure 6:**
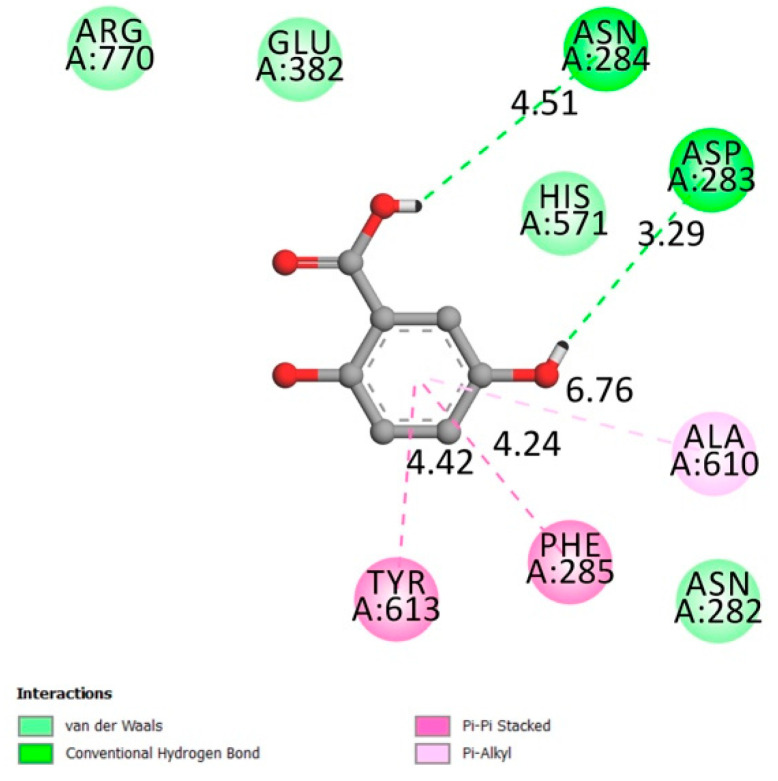
Two-dimensional scheme of the GA interactions with glycogen phosphorylase receptor.

The inhibition of the enzyme are controlled by the amino acids 280–288 (The 280’s loop) [43,44]. Figure 6 confirms the interaction of gentisic acid with GP’s 280’s loop with an affinity of −6.3 kcal/mol. 

Glycogen phosphorylase is known to be one of the causes of hyperglycemia since it is responsible for the development of glucose. Inhibiting this can contribute to a substantial improvement in the glycemic condition of diabetic people.

The interactions, even when established with the receptors on their active site, did not make a sign of a remarkable activity as the highest affinity reached only −6.9 kcal/mol with the aldose reductase receptor compared to amentoflavone a well-known antidiabetic molecule who exhibited very excellent affinities to almost all receptors (−8.8 to −11.3 kcal/mol). The docking results reading and, when compared to other studies, didn’t give any sign of a solid and performed interaction [45]. The ligand binds with most of the receptors tested but did not act in a way to make it a potential antidiabetic molecule. The results presented will ensure a better understanding of the key activities of gentisic acid and to help identify the molecule’s true action in a given plant extract or molecule mixture.

Phenolic acids, which are naturally produced by numerous plants, are assumed to have a strong antioxidant and free radical scavenging ability, with the mechanism of inhibiting the enzymes that generate ROS [46]. Latest study has shown that oxidative stress exacerbates the development of diabetes, while consuming an antioxidant-rich diet decreases it risk [47]. Gentisic acid did not demonstrate an oriented inhibition toward a specific receptor but it general ability as antioxidant and anti-inflammatory [9] could be beneficial as a treatment complement for people with diabetes; in contrast, amentoflavone with its spectacular affinities toward multiple receptors could serve as a specific inhibitor such as DPP4 or α-glucosidase inhibitors which are a class of antidiabetic commercialized drugs. 

### 2.2. In Vitro Assays

The inhibition of glucose absorption is one of the efficient strategies used for treating diabetes. By inhibiting the digestive enzymes responsible for polysaccharides’ hydrolysis into tiny absorbable fragments, we prevent postprandial elevated blood glucose [48]. Among those enzymes are α-glucosidase and α-amylase. Gentisic acid will be evaluated against these two enzymes for a possible inhibitory effect to identify one or more of this molecule mode of action

#### 2.2.1. Inhibitory Effect of α-Amylase

α-amylase is known to be one of the most important enzymes in the digestive process. Predominantly present in saliva and pancreatic juice, because of its significant function in the breakdown of polysaccharides [30]. One of the potential strategies for avoiding elevated postprandial blood glucose is to target and suppress this enzyme [37]. 

**Figure 7:**
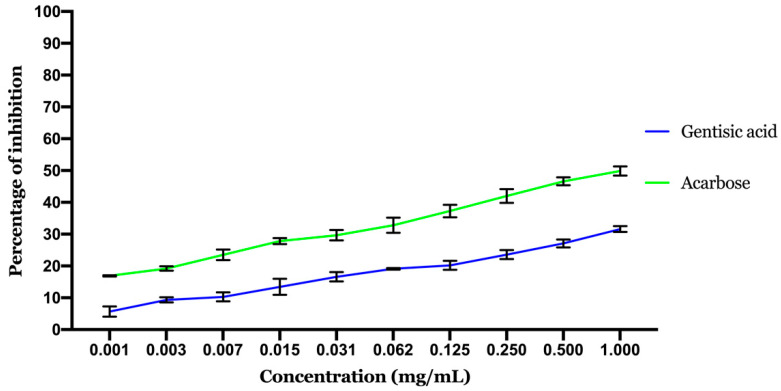
Results of α-amylase inhibitory activity. Values are expressed as mean ± standard deviation (SD, *n* = 3).

α-amylase inhibition potential of gentisic acid is displayed in Figure 7. The inhibition of the enzyme tends to be related to the dosage, as the concentration of the GA clearly influences the amount of enzyme inhibited. The calculated IC50 (concentration needed to inhibit 50% of the enzyme activity) showed that acarbose (positive control) inhibition potential is better than GA with an IC50 of 0.717 ± 0.054 mg/mL noted for acarbose against 2.07 ± 0.3 mg/mL noted for GA.

GA exhibited a very moderate activity compared to acarbose in total agreement with the in silico results above.

#### 2.2.2. Inhibitory Effect of α-Glucosidase

The α-glucosidase enzyme, found in the mucosal brush boundary of the small intestine, is also one of the main digestion enzymes. Its role is to transform and degrade complex carbohydrates into short, quick and absorbable carbohydrates. Inhibition is an important way to slow the absorption of glucose and also to avoid elevated levels of postprandial blood glucose, which may slow the development of diabetes.

**Figure 8:**
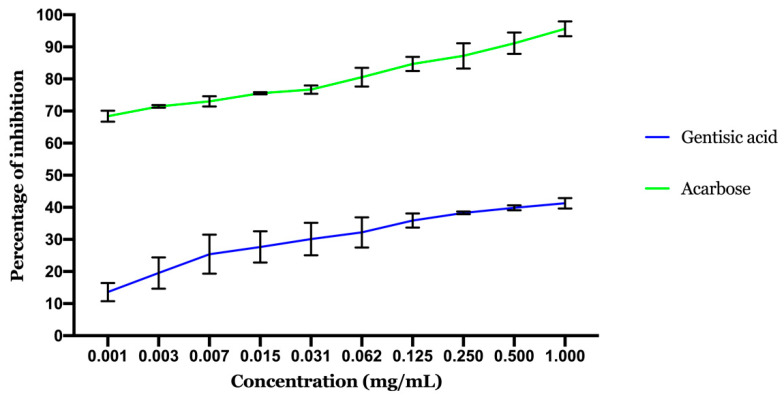
Results of α-glucosidase inhibitory activity. Values are expressed as mean ± SD (*n* = 3).

Figure 8 displays the α-glucosidase inhibition activity of gentisic acid. As the highest concentrations exhibited the highest inhibition activity, the inhibition effect is correlated to the GA concentration. The calculated IC50 showed that the inhibition potential of acarbose (IC50 0.00084 ± 0.00007 mg/mL) is better than GA (IC50 1.69 ± 0.027 mg/mL). GA activity was also in accordance with the docking results with moderate activity.

## 3. Materials and Methods

### 3.1. Chemicals and Reagents

Gentisic acid (CAS number: 490-79-9; MW: 154.12), α-amylase (CAS number: 9000-85-5) α-glucosidase (CAS number: 9001-42-7) were procured from Sigma-Aldrich (St. Louis, MO, USA). The other chemicals and reagents used in this study were of analytical grade.

### 3.2. Molecular Docking 

#### 3.2.1. Ligand Preparation 

The gentisic acid SDF file was obtained from PubChem (CID: 3469) and then converted to the PDBQT format with AutoDockTools v1.5.6 [49] for the docking simulation. The preparation included the addition of the Gasteiger partial charges, the definition of rotatable bonds, and the merge of the non-polar hydrogen atoms.

#### 3.2.2. Receptors’ Preparation 

The PDB file of each receptor was retrieved from the protein data bank website (https://www.rcsb.org, accessed on 25 March 2021) [50]. Precise X-ray crystal structures of the receptors were chosen based on their completeness, resolution, and their fit with our objective. The selected receptors (PID) were as follows: PTP1B (1c83), glycogen phosphorylase (1l5q), FFAR1 (4phu), PPAR gamma, (5ycp), α-amylase (1smd), α-glucosidase (5nn5), aldose reductase (2hv5) and DPP4 (2p8s).

Using the windows software Discovery Studio Visualizer v19.1.0, the receptors were prepared by removing water molecules, the default ligand, and heteroatoms. The modified receptors were opened with AutoDockTools to add polar hydrogen atoms, Gasteiger charges and were converted into PDBQT for further docking simulation.

#### 3.2.3. Docking Simulations

The grid box size was specified for each receptor using AutoDock Tools, and AutoDock Vina [51] was used for carrying out the docking simulations for the gentisic acid (Ligand) and the eight receptors. The simulation exhaustiveness was set to 24 to ensure Vina provided the best results possible within a reasonable time period. The protein-ligand complexes visualization was carried out using Discovery Studio Visualizer V19. 

### 3.3. Enzymes In Vitro Inhibition

#### 3.3.1. Assay of the α-Amylase Inhibitory Effect

The assay was performed following Mitra et al. protocol [52]. A 0.2 mL solution of 0.5M Tris-HCl buffer (pH 6.9) containing 0.01M CaCl_2_ was mixed with 2 mg of starch to prepare the substrate solution. The substrate solution was spread into test tubes, boiled (for 5 min), and pre-incubated (for 5 min at 37 °C). GA was dissolved and prepared at varying concentrations of 1, 3, 7, 15, 31, 62, 125, 250, 500 and 1000 μg/mL using DMSO. 0.2 mL of the GA solution (0.2 mL) was added at various concentrations was added to the substrate solution, and then, 0.1 mL of alpha amylase (2 units per mL in Tris–HCl buffer) was added. This reaction was carried out for 10 min at 37 °C and then halted by applying 0.5 mL of 50% acetic acid to each test tube. Centrifugation was undertaken (3000 rpm at 4 °C for 5 min), and the optical density of the supernatant was performed at 595 nm using a spectrophotometer. Acarbose (α-amylase inhibitor) was used as a positive control in this assay. For each concentration, the tests were replicated thrice and represented in terms of mean values and standard deviation values (X ± SD).

The inhibitory action of α-amylase was estimated using the following formula:

Inhibitory action of α-amylase = [(X − Y)/X] × 100.(1)

X is the control absorbance, and Y is the sample absorbance.

The IC50 values for acarbose and GA (concentration needed to inhibit 50 percent of the enzyme activity) were calculated after evaluating the α-amylase inhibitory activity of the various concentrations.

#### 3.3.2. Assay of the α-Glucosidase Inhibitory Effect

The test was conducted using the procedure of Pistia Brueggeman and Hollingsworth [53]. At different concentrations (1, 3, 7, 15, 31, 62, 125, 250, 500 and 1000 μg/mL), 50 μL of GA was prepared and incubated with a solution comprising 10 μL of α-glucosidase 1 U/mL and 125 μL of 0.1 M phosphate buffer (pH 6.8) at 37 °C for 20 min. A solution of 20 μL of 1 M pNPG (substrate) was used to initiate the reaction and then incubated for half an hour. We applied 50 μL of 0.1 N Na_2_CO_3_ to stop the reaction. The optical density was calculated by a spectrophotometer at 405 nm. Acarbose (α-glucosidase inhibitor) was used as a positive control in this assay. For each concentration, the tests were performed three times (*n* = 3) and represented in terms of mean values and standard deviation values (X ± SD). The α-glucosidase inhibitory activity was calculated by using the following formula:

Inhibitory action of α-glucosidase = [(X − Y)/X] × 100.(2)

X is the control absorbance, and Y is the sample absorbance.

The IC50 values for acarbose and GA (concentration needed to inhibit 50% of the enzyme activity) were calculated after evaluating the α-amylase inhibitory activity of the various concentrations.

## 4. Conclusions

Despite its multiple pharmacological activities, and besides its interaction with most of the receptors tested, the antidiabetic activity of gentisic acid was moderate and did not indicate any real potential in the frame of the investigation performed in this research work. This conclusion is partially demonstrated by the in vitro tests, which confirm the α-amylase and α-glucosidase’s in silico results. This research was conducted in a scientific context to discover and study a plant-based bioactive molecule (gentisic acid) in a manner to approve or deny its potential antidiabetic activity for a better understanding of this molecule’s overall action on the human body as it is present in many daily consumed foods. Although the in vitro and in silico studies were conducted, an in vivo study should be performed to confirm these results.

## Figures and Tables

**Table 1:** Overview of affinities of gentisic acid to receptors.

	Affinity (kcal/mol)							
Molecule	PTP1B	DPP4	FFAR1	α-amylase	PPAR Gamma	α-glucosidase	Aldose Reductase	Glycogen Phosphorylase
Gentisic acid	−6.1	−6.7	None	−5.6	None	−6.1	−6.9	−6.3
Amentoflavone	−8.8	−10.5	None	−11.3	None	−9.5	−10.0	−10.7

## Data Availability

Data are available upon reasonable request.

## Supplementary Materials

The following are available online, Figure S1: 2D schemes of Amentoflavone interactions with the different receptors.

## Sample Availability

Samples of the compounds are available from the authors upon reasonable request.

## References

[1] Es-Safi I., Mechchate H., Amaghnouje A., Jawhari F.Z., Bari A., Cerruti P., Avella M., Andriy A., Andriy D. (2020). Medicinal Plants Used to Treat Acute Digestive System Problems in the Region of Fez-Meknes in Morocco: An Ethnopharmacological Survey. Ethnobot. Res. Appl..

[2] Parveen A., Parveen B., Parveen R., Ahmad S. (2015). Challenges and Guidelines for Clinical Trial of Herbal Drugs. J. Pharm. Bioallied Sci..

[3] Atanasov A.G., Waltenberger B., Pferschy-Wenzig E.-M., Linder T., Wawrosch C., Uhrin P., Temml V., Wang L., Schwaiger S., Heiss E.H. (2015). Discovery and Resupply of Pharmacologically Active Plant-Derived Natural Products: A Review. Biotechnol. Adv..

[4] Süntar I. (2020). Importance of Ethnopharmacological Studies in Drug Discovery: Role of Medicinal Plants. Phytochem. Rev..

[5] Thomford N., Senthebane D., Rowe A., Munro D., Seele P., Maroyi A., Dzobo K. (2018). Natural Products for Drug Discovery in the 21st Century: Innovations for Novel Drug Discovery. IJMS.

[6] Kiokias S., Proestos C., Oreopoulou V. (2020). Phenolic Acids of Plant Origin—A Review on Their Antioxidant Activity In Vitro (O/W Emulsion Systems) Along with Their in Vivo Health Biochemical Properties. Foods.

[7] Russell W., Duthie G. (2011). Plant Secondary Metabolites and Gut Health: The Case for Phenolic Acids. Proc. Nutr. Soc..

[8] Morse J.M. (1995). The Significance of Saturation. Qual. Health. Res..

[9] Abedi F., Razavi B.M., Hosseinzadeh H. (2020). A Review on Gentisic Acid as a Plant Derived Phenolic Acid and Metabolite of Aspirin: Comprehensive Pharmacology, Toxicology, and Some Pharmaceutical Aspects. Phytother. Res..

[10] Shivanagoudra S.R., Perera W.H., Perez J.L., Athrey G., Sun Y., Wu C.S., Jayaprakasha G.K., Patil B.S. (2019). In Vitro and in Silico Elucidation of Antidiabetic and Anti-Inflammatory Activities of Bioactive Compounds from *Momordica charantia* L.. Bioorg. Med. Chem..

[11] Mullen W., Edwards C.A., Crozier A. (2006). Absorption, Excretion and Metabolite Profiling of Methyl-, Glucuronyl-, Glucosyl- and Sulpho-Conjugates of Quercetin in Human Plasma and Urine after Ingestion of Onions. Br. J. Nutr..

[12] Su C., Yang C., Gong M., Ke Y., Yuan P., Wang X., Li M., Zheng X., Feng W. (2019). Antidiabetic Activity and Potential Mechanism of Amentoflavone in Diabetic Mice. Molecules.

[13] Elchebly M. (1999). Increased Insulin Sensitivity and Obesity Resistance in Mice Lacking the Protein Tyrosine Phosphatase-1B Gene. Science.

[14] Goldstein B.J. (2001). Protein-Tyrosine Phosphatase 1B (PTP1B): A Novel Therapeutic Target for Type 2 Diabetes Mellitus, Obesity and Related States of Insulin Resistance. Curr. Drug Targets Immune Endocr. Metabol. Disord..

[15] Klaman L.D., Boss O., Peroni O.D., Kim J.K., Martino J.L., Zabolotny J.M., Moghal N., Lubkin M., Kim Y.-B., Sharpe A.H. (2000). Increased Energy Expenditure, Decreased Adiposity, and Tissue-Specific Insulin Sensitivity in Protein-Tyrosine Phosphatase 1B-Deficient Mice. Mol. Cell. Biol..

[16] Jia Z., Barford D., Flint A., Tonks N. (1995). Structural Basis for Phosphotyrosine Peptide Recognition by Protein Tyrosine Phosphatase 1B. Science.

[17] Johnson T.O., Ermolieff J., Jirousek M.R. (2002). Protein Tyrosine Phosphatase 1B Inhibitors for Diabetes. Nat. Rev. Drug Discov..

[18] Wiesmann C., Barr K.J., Kung J., Zhu J., Erlanson D.A., Shen W., Fahr B.J., Zhong M., Taylor L., Randal M. (2004). Allosteric Inhibition of Protein Tyrosine Phosphatase 1B. Nat. Struct. Mol. Biol..

[19] Röhrborn D. (2015). DPP4 in Diabetes. Front. Immunol..

[20] Matteucci E., Giampietro O. (2009). Dipeptidyl Peptidase-4 (CD26): Knowing the Function before Inhibiting the Enzyme. CMC.

[21] Bjelke J.R., Christensen J., Branner S., Wagtmann N., Olsen C., Kanstrup A.B., Rasmussen H.B. (2004). Tyrosine 547 Constitutes an Essential Part of the Catalytic Mechanism of Dipeptidyl Peptidase IV. J. Biol. Chem..

[22] Chien C.-H., Tsai C.-H., Lin C.-H., Chou C.-Y., Chen X. (2006). Identification of Hydrophobic Residues Critical for DPP-IV Dimerization. Biochemistry.

[23] Ren X.-M., Cao L.-Y., Zhang J., Qin W.-P., Yang Y., Wan B., Guo L.-H. (2016). Investigation of the Binding Interaction of Fatty Acids with Human G Protein-Coupled Receptor 40 Using a Site-Specific Fluorescence Probe by Flow Cytometry. Biochemistry.

[24] Srivastava A., Yano J., Hirozane Y., Kefala G., Gruswitz F., Snell G., Lane W., Ivetac A., Aertgeerts K., Nguyen J. (2014). High-Resolution Structure of the Human GPR40 Receptor Bound to Allosteric Agonist TAK-875. Nature.

[25] Morgan N.G., Dhayal S. (2009). G-Protein Coupled Receptors Mediating Long Chain Fatty Acid Signalling in the Pancreatic Beta-Cell. Biochem. Pharmacol..

[26] Sum C.S., Tikhonova I.G., Costanzi S., Gershengorn M.C. (2009). Two Arginine-Glutamate Ionic Locks Near the Extracellular Surface of FFAR1 Gate Receptor Activation. J. Biol. Chem..

[27] Sum C.S., Tikhonova I.G., Neumann S., Engel S., Raaka B.M., Costanzi S., Gershengorn M.C. (2007). Identification of Residues Important for Agonist Recognition and Activation in GPR40. J. Biol. Chem..

[28] Suvd D., Fujimoto Z., Takase K., Matsumura M., Mizuno H. (2001). Crystal Structure of Bacillus Stearothermophilus α-amylase: Possible Factors Determining the Thermostability. J. Biochem..

[29] Aghajari N., Feller G., Gerday C., Haser R. (1998). Crystal Structures of the Psychrophilic α-amylase from Alteromonas Haloplanctis in Its Native Form and Complexed with an Inhibitor. Protein Sci..

[30] Hsiu J., Fischer E.H., Stein E.A. (1964). α-amylases As Calcium-Metalloenzymes. II. Calcium and the catalytic activity. Biochemistry.

[31] Ragunath C., Manuel S.G.A., Venkataraman V., Sait H.B.R., Kasinathan C., Ramasubbu N. (2008). Probing the Role of Aromatic Residues at the Secondary Saccharide-Binding Sites of Human Salivary α-Amylase in Substrate Hydrolysis and Bacterial Binding. J. Mol. Biol..

[32] Ramasubbu N., Ragunath C., Sundar K., Mishra P.J., Gyémánt G., Kandra L. (2005). Structure-Function Relationships in Human Salivary α-Amylase: Role of Aromatic Residues. Biol. Sect. Cell. Mol. Biol..

[33] Ramasubbu N., Ragunath C., Mishra P.J., Thomas L.M., Gyemant G., Kandra L. (2004). Human Salivary α-amylase Trp58 Situated at Subsite -2 Is Critical for Enzyme Activity. Eur. J. Biochem..

[34] Ahmadian M., Suh J.M., Hah N., Liddle C., Atkins A.R., Downes M., Evans R.M. (2013). PPARγ Signaling and Metabolism: The Good, the Bad and the Future. Nat. Med..

[35] Pochetti G., Godio C., Mitro N., Caruso D., Galmozzi A., Scurati S., Loiodice F., Fracchiolla G., Tortorella P., Laghezza A. (2007). Insights into the Mechanism of Partial Agonism: Crystal structures of the peroxisome proliferator-activated receptor γ ligand-binding domain in the complex with two enantiomeric ligands. J. Biol. Chem..

[36] Jiang J., Ghosh S. (2019). Glucosidase. RCSB Protein Data Bank.

[37] Alqahtani A.S., Hidayathulla S., Rehman M.T., ElGamal A.A., Al-Massarani S., Razmovski-Naumovski V., Alqahtani M.S., El Dib R.A., AlAjmi M.F. (2019). α-amylase and α-glucosidase Enzyme Inhibition and Antioxidant Potential of 3-Oxolupenal and Katononic Acid Isolated from Nuxia Oppositifolia. Biomolecules.

[38] Hermans M.M., Kroos M.A., van Beeumen J., Oostra B.A., Reuser A.J. (1991). Human Lysosomal α-glucosidase. Characterization of the Catalytic Site. J. Biol. Chem..

[39] Tang W.H., Martin K.A., Hwa J. (2012). Aldose Reductase, Oxidative Stress, and Diabetic Mellitus. Front. Pharmacol..

[40] Heather L.C., Clarke K. (2011). Metabolism, Hypoxia and the Diabetic Heart. J. Mol. Cell. Cardiol..

[41] Wilson D.K., Bohren K.M., Gabbay K.H., Quiocho F.A. (1992). An Unlikely Sugar Substrate Site in the 1.65 A Structure of the Human Aldose Reductase Holoenzyme Implicated in Diabetic Complications. Science.

[42] Livanova N.B., Chebotareva N.A., Eronina T.B., Kurganov B.I. (2002). Pyridoxal 5′-Phosphate as a Catalytic and Conformational Cofactor of Muscle Glycogen Phosphorylase B. Biochemistry.

[43] Barford D., Johnson L.N. (1989). The Allosteric Transition of Glycogen Phosphorylase. Nature.

[44] Goldsmith E., Sprang, Hamlin R., Xuong N., Fletterick R. (1989). Domain Separation in the Activation of Glycogen Phosphorylase A. Science.

[45] Mechchate H., Es-Safi I., Bourhia M., Kyrylchuk A., El Moussaoui A., Conte R., Ullah R., Ezzeldin E., Mostafa G.A., Grafov A. (2020). In-Vivo Antidiabetic Activity and In-Silico Mode of Action of LC/MS-MS Identified Flavonoids in Oleaster Leaves. Molecules.

[46] Thyagaraju B.M. (2008). Muralidhara Ferulic Acid Supplements Abrogate Oxidative Impairments in Liver and Testis in the Streptozotocin-Diabetic Rat. Zool. Sci..

[47] Patel S.S., Goyal R.K. (2011). Cardioprotective Effects of Gallic Acid in Diabetes-Induced Myocardial Dysfunction in Rats. Pharmacogn. Res..

[48] Min S.W., Han J.S. (2014). Polyopes Lancifolia Extract, a Potent α-Glucosidase Inhibitor, Alleviates Postprandial Hyperglycemia in Diabetic Mice. Prev. Nutr. Food Sci..

[49] Morris G.M., Huey R., Lindstrom W., Sanner M.F., Belew R.K., Goodsell D.S., Olson A.J. (2009). AutoDock4 and AutoDockTools4: Automated Docking with Selective Receptor Flexibility. J. Comput. Chem..

[50] Berman H.M., Battistuz T., Bhat T.N., Bluhm W.F., Bourne P.E., Burkhardt K., Feng Z., Gilliland G.L., Iype L., Jain S. (2002). The Protein Data Bank. Acta Crystallogr. Sect. D Biol. Crystallogr..

[51] Trott O., Olson A.J. (2009). AutoDock Vina: Improving the Speed and Accuracy of Docking with a New Scoring Function, Efficient Optimization, and Multithreading. J. Comput. Chem..

[52] Mitra A., Tamil I., Dineshkumar B., Nandhakumar M., Senthilkumar M. (2010). In Vitro Study on α-Amylase Inhibitory Activity of an Indian Medicinal Plant, Phyllanthus Amarus. Indian J. Pharmacol..

[53] Pistia-Brueggeman G., Hollingsworth R.I. (2001). A Preparation and Screening Strategy for Glycosidase Inhibitors. Tetrahedron.

