# Supplementary material for: Insight into Gentisic Acid Antidiabetic Potential Using In Vitro and In Silico Approaches
Source: Molecules. 2021 Mar 30;26(7):1932. doi: 10.3390/molecules26071932 (PMC8037080; doi:10.3390/molecules26071932)
Supplement: Supplementary file 1 [file molecules-26-01932-s001.pdf]

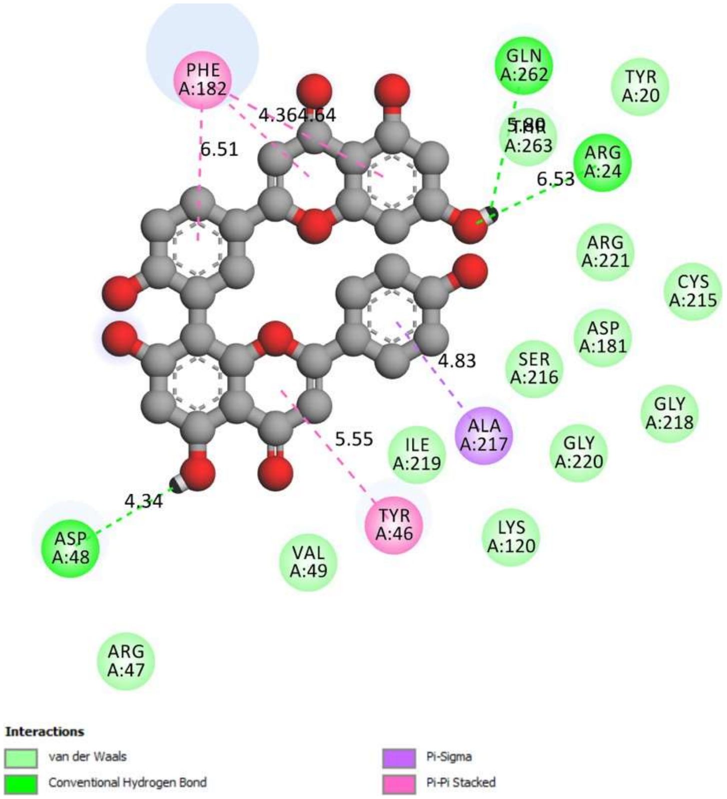

Figure S1. Amentoflavone interaction with the PTB1B receptor

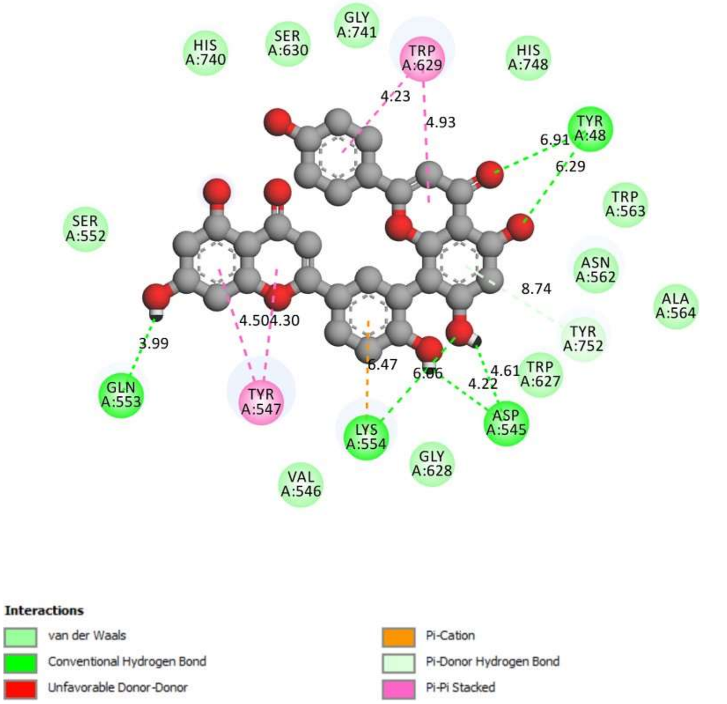

Figure S2. Amentoflavone interaction with the DPP4 receptor

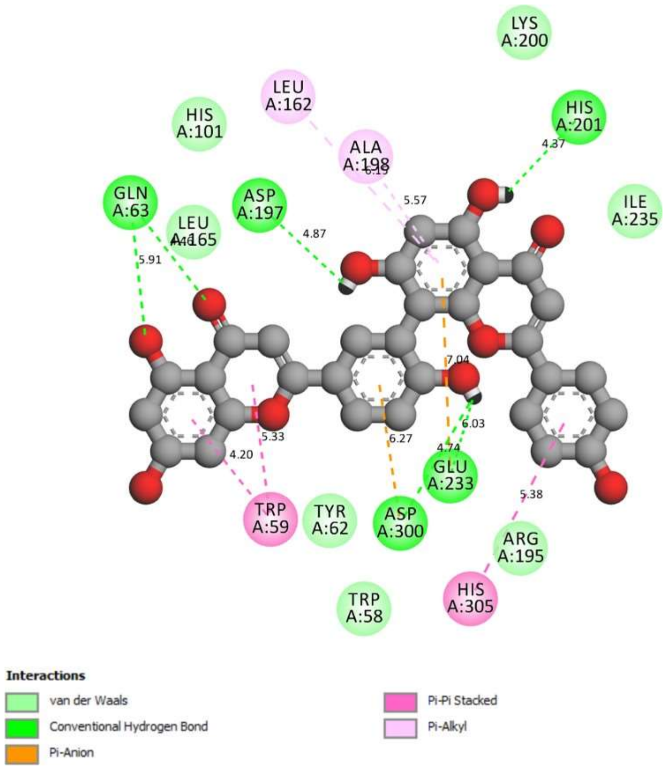

Figure S3. Amentoflavone interaction with the Alpha amylase receptor

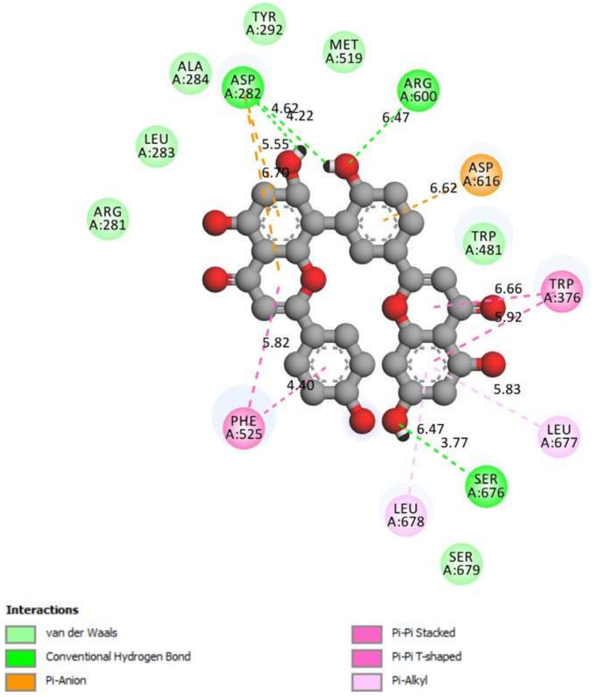

Figure S4. Amentoflavone interaction with the Alpha glucosidase receptor

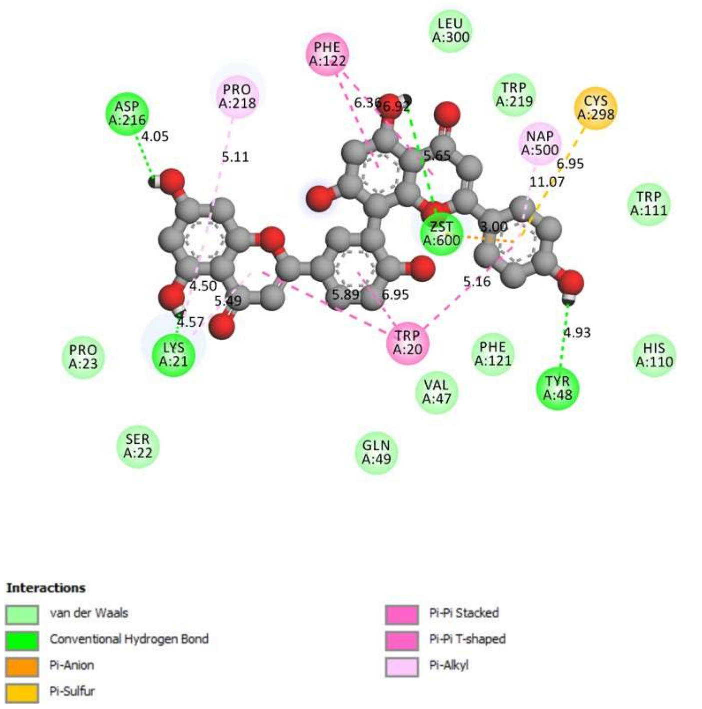

Figure S5. Amentoflavone interaction with the Aldose reductase receptor

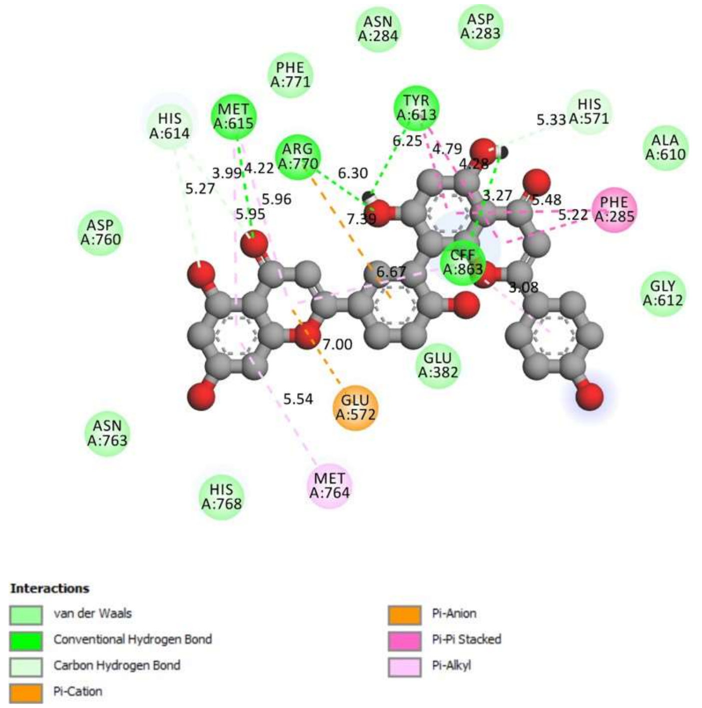

Figure S6. Amentoflavone interaction with the Glycogen phosphorylase receptor
